# Insight Into the Pico- and Nano-Phytoplankton Communities in the Deepest Biosphere, the Mariana Trench

**DOI:** 10.3389/fmicb.2018.02289

**Published:** 2018-09-26

**Authors:** Ruoyu Guo, Yantao Liang, Yu Xin, Long Wang, Shanli Mou, Chunjie Cao, Ruize Xie, Chuanlun Zhang, Jiwei Tian, Yongyu Zhang

**Affiliations:** ^1^Key Laboratory of Biofuels, Shandong Provincial Key Laboratory of Energy Genetics, Qingdao Institute of Bioenergy and Bioprocess Technology, Chinese Academy of Sciences, Qingdao, China; ^2^Physical Oceanography Laboratory/Qingdao Collaborative Innovation Center of Marine Science and Technology, Key Laboratory of Marine Chemistry Theory & Engineering, Ocean University of China, Qingdao, China; ^3^Department of Ocean Science and Engineering, Southern University of Science and Technology, Shenzhen, China

**Keywords:** pico- and nano-phytoplankton, 18S rRNA, plastid 23S rRNA, Mariana Trench, deep sea

## Abstract

As photoautotrophs, phytoplankton are generally present in the euphotic zone of the ocean, however, recently healthy phytoplankton cells were found to be also ubiquitous in the dark deep sea, i.e., at water depths between 2000 and 4000 m. The distributions of phytoplankton communities in much deeper waters, such as the hadal zone, are unclear. In this study, the vertical distribution of the pico- and nano-phytoplankton (PN) communities from the surface to 8320 m, including the epipelagic, mesopelagic, bathypelagic, and hadal zones, were investigated via both 18S and p23S rRNA gene analysis in the Challenger Deep of the Mariana Trench. The results showed that Dinoflagellata, Chrysophyceae, Haptophyta, Chlorophyta, Prochloraceae, Pseudanabaenaceae, Synechococcaceae, and Eustigmatophyceae, etc., were the predominant PN in the Mariana Trench. Redundancy analyses revealed that depth, followed by temperature, was the most important environmental factors correlated with vertical distribution of PN community. In the hadal zone, the PN community structure was considerably different from those in the shallower zones. Some PN communities, e.g., Eustigmatophyceae and Chrysophyceae, which have the heterotrophic characteristics, were sparse in shallower waters, while they were identified with high relative abundance (94.1% and 20.1%, respectively) at the depth of 8320 m. However, the dinoflagellates and Prochloraceae *Prochlorococcus* were detected throughout the entire water column. We proposed that vertical sinking, heterotrophic metabolism, and/or the transition to resting stage of phytoplankton might contribute to the presence of phytoplankton in the hadal zone. This study provided insight into the PN community in the Mariana Trench, implied the significance of phytoplankton in exporting organic matters from the euphotic to the hadal zone, and also hinted the possible existence of some undetermined energy metabolism (e.g., heterotrophy) of phytoplankton making themselves adapt and survive in the hadal environment.

## Introduction

Oceans represent the world’s largest ecosystem, which comprise diverse visible and invisible organisms that directly or indirectly support carbon cycling in the global biosphere. The hadal trench, which is deeper than 6000 m below the sea surface, represents the most pressurized aquatic environment (Jamieson et al., 2010; Nunoura et al., 2015). Many organisms, such as metazoans, protozoans, bacteria, and archaea, have been identified from the hadal trench, and previous results have shown that the genetic characteristics and community structures of organisms in the hadal zone were differentiated from those in shallower waters or the abyssal zone (Kato et al., 1997; Takami et al., 1997; Vinogradova, 1997; Nunoura et al., 2015; Jonathan et al., 2016). The unique environmental factors, such as geomorphology and hydrostatic pressure, may contribute to shaping the distinct assemblage structure of organisms in the hadal trench (Nunoura et al., 2015; Jonathan et al., 2016). The distinct phenotypical and genetic features of the metazoans, protozoans, and microbes that inhabit the hadal zone (Danovaro et al., 2002; Tamburini et al., 2013; Lan et al., 2017) may be optimized for adapting to the extreme environmental conditions.

Phytoplankton are a tremendously diverse group of single-celled bacteria and eukaryotes that are the base of food web in the ocean ecosystem, and are the most important primary producer that could be responsible for approximately 50% of the net primary production of the global biosphere (Field et al., 1998; Halsey and Jones, 2015). They could affect the abundance and diversity of marine organisms (Chassot et al., 2010; Buchan et al., 2014) and biogeochemical cycling (Behrenfeld et al., 2005; Richardson and Jackson, 2007; Lomas et al., 2014; Marañón, 2015; Leblanc et al., 2018). Picophytoplankton (0.2–2 or 3 μm) and nanophytoplankton (2 or 3–20 μm) are ubiquitous and principal components of phytoplankton biomass and make an important contribution to primary production in the ocean (Detmer and Bathmann, 1997; Kaiser et al., 2007; Vaulot et al., 2008; Wang et al., 2010; Liang et al., 2017), and these small phytoplankton also play important roles in biogeochemical cycling such as contributing to carbon export from the surface ocean (Richardson and Jackson, 2007; Tang et al., 2014; Leblanc et al., 2018). To date, most of phytoplankton study focus on the shallower waters, little is known about the phytoplankton in the deep sea especially in the hadal zone. However, several studies have identified the phytoplankton cells and phytodetritus from the deep sea as well as the hadal trench. Healthy phytoplankton cells have been found at water depths of 2000–4000 m in the world’s oceans (Agusti et al., 2015). *Prochlorococcus*, which is the smallest autotroph in the ocean, has been identified in the Challenger Deep of the Mariana Trench (Jonathan et al., 2016), and algal pigments, such as phaeophytin and chlorophyll *a*, have also been identified in the hadal trench and in the sediments (Glud et al., 2013). Latest study revealed that algae might be the primary source of organic matter at the Challenger Deep and in the hadal trench as well (Luo et al., 2017). Nevertheless, the diversity and composition of phytoplankton in the hadal trench is still unclear.

In recent years, molecular methods have been widely employed to study phytoplankton communities in various environmental conditions (Shi et al., 2011; Treusch et al., 2012; Kirkham et al., 2013; Agusti et al., 2015; Balzano et al., 2015; Liu et al., 2015). These molecular methods generally include the amplicon sequencing-based detection technique. The choice of the proper primer is one of the most important factors that affect the results. The ribosomal DNA genes have become standard target genes to investigate the community structure and diversity of microbes. For phytoplankton, several primers that target plastid 23S rRNA (p23S rRNA), plastid 16S rRNA (p16S rRNA), and 18S rRNA have been employed to investigate the community structure (Sherwood and Presting, 2007; Treusch et al., 2012; Bazin et al., 2014). For a comprehensive understanding of the phytoplankton composition, a combination of analyses using several primers has been suggested as a better solution (Shi et al., 2011).

The Challenger Deep of the Mariana Trench in the Northwest Pacific is the deepest biosphere on Earth, with a water depth of 10,920 m (Nakanishi and Hashimoto, 2011). The physical and chemical characteristics, such as geomorphology, temperature, salinity, current patterns, oxygen consumption, organic matter (Fujioka et al., 2002; Taira et al., 2004; Taira et al., 2005; Glud et al., 2013; Luo et al., 2017), and microbial community diversity and composition, have been investigated (Nunoura et al., 2015; Jonathan et al., 2016). Nevertheless, knowledge about the phytoplankton diversity in the Mariana Trench is very limited. In this study, we conducted 18S and p23S rRNA gene surveys on pico- and nano-phytoplankton (PN) communities (0.22 μm ≤ size < 20 μm). The vertical distribution of PN communities in the epipelagic, mesopelagic, bathypelagic, and hadal zones of the Mariana Trench were investigated. The data illustrated key environmental factors that regulate the vertical distribution patterns of the PN communities in the Mariana Trench and potential reasons of the presence of phytoplankton in the dark sea.

## Materials and Methods

### Sampling and Geochemical Analyses

Water samples were collected at the station Challenge Deep from 11 layers of the Mariana Trench (See sample information, **Supplementary Table S1**) in March 2017. Seawater samples from 4 to 4000 m were collected by Niskin Bottle mounted to a Seabird conductivity, temperature, and depth (CTD; SBE 25, Sea-Bird Co.); whereas water samples at depth of 6050 and 8320 m were collected by a self-designed acoustic-controlled full ocean depth water sampler. Five-liter of seawater from each layer was firstly filtered using 20-μm pore-size membrane filters, and then the subsamples were harvested onto 0.22-μm pore-size membrane filters (Millipore, Germany). The 0.22-μm pore-size membrane filters were used in the further analysis. A total of two filters (duplicates) were prepared for each seawater sample. The filters were frozen immediately in liquid nitrogen and kept at -20°C on board. By the end of cruise, the filters were transferred to -80°C refrigerator in land-based lab until DNA extraction. The environmental parameters (i.e., depth, temperature, and salinity) were measured using a CTD sensor (SBE 25, Sea-Bird Co.). Nutrients and dissolved oxygen (DO) were sampled and analyzed following the protocols described in Grasshoff et al. (1999).

### Cell Abundance Measurement

Heterotrophic prokaryote and picoeukaryote abundances were determined on a BD FACSAria II flow cytometer (BD Biosciences, CA, United States). The procedure and analysis followed the protocol described by Liang et al. (2016). For the picoeukaryote abundances, only the autotrophic picoeukaryote was included in the analysis.

### DNA Extraction and Sequencing

Total DNA was extracted following the method described by Boopathi et al. (2015). DNA quantity and integrity were verified using NanoDrop (Thermo-Fisher, United States) and 1% (w/v) agarose gel. To investigate the PN composition, two gene markers including nuclear 18S rRNA and p23S rRNA gene were employed. The 18S rRNA gene can provide information on the eukaryotic PN composition, and the p23S rRNA gene can be used to analyze the community composition of cyanobacteria and eukaryotic algae (Presting, 2006). The V1–V3 region of the eukaryotic 18S rRNA gene was amplified with the primers 82F (5′-GAAACTGCGAATGGCTC-3′) (López-García et al., 2003) and 516R (5′-ACCAGACTTGCCCTCC-3′) (Casamayor et al., 2002). For plastid 23S rRNA gene analysis, the primers p23SrV_f1 (5′-GGA CAG AAA GAC CCT ATG AA-3′) and p23SrV_r1 (5′ TCA GCC TGT TAT CCC TAG AG-3′) (Presting, 2006) were used in the PCR amplification. Equimolar amounts of each sample were mixed and sequenced using Illumina MiSeq (Illumina Inc., United States). The sequencing was conducted at Majorbio Bio-Pharm Technology Co., Ltd. (Shanghai, China). The two filters from each layer were used for DNA extraction and sequencing, respectively. All the sequence data generated in this study have been deposited in NCBI Sequence Read Archive database under the accession number SRP154062.

### Sequence Processing, Operational Taxonomic Unit (OTU) Assignment, and Environmental Factor Analyses

The raw data were filtered to eliminate low-quality reads to obtain clean reads. The raw data trimming and assembly were performed using Trimmomatic and FLASH v1.2.11, respectively (Magoc and Salzberg, 2011; Bolger et al., 2014). The reads were assembled with a minimum overlap length of 10 bp. All reads that completely matched the barcodes were retained, as were reads with a maximum 2 bp mismatch from the primers. Then, the primers and barcodes were trimmed from the sequences. The sequences were clustered into OTUs at a similarity of 97% using USEARCH (version 7.0) (Edgar, 2010).

For the taxonomic annotation, the representative 18S rRNA gene OTU sequences were annotated by comparison against the SILVA database with a similarity cutoff value of 0.7 (Quast et al., 2013). The p23S rRNA gene sequences were annotated by BLASTn alignment against the NCBI non-redundant nucleotide (NT) database. Taxonomic classifications for each OTU (similarity ≥97%) were obtained using the RDP Classifier. According to taxonomic annotation of 18S and p23S rRNA gene, only the OTUs that assigned to phytoplankton were employed in further analysis by removing OTUs assigned to opisthokonta, heterotrophic bacteria, and so on. The Chao1 richness (Chao and Bunge, 2002) and Shannon diversity index (Magurran, 1989) values were calculated using MOTHUR version v.1.30.1 (Schloss et al., 2011). All of the mentioned analyses above were carried out on the free online platform of Majorbio I-Sanger Cloud Platform^1^. The taxonomy classification was described according to the taxonomic description of NCBI taxonomy database. Generally, the phylum level was used as classification for eukaryotic phytoplankton. However, some phytoplankton belonged to stramenopiles (no rank), in this case, the class level were employed for taxonomy description. As for the cyanobacteria, all the identified OTUs belonged to cyanobacteria Synechococcales (order; no classification at class level), the family level were employed to distinguish them. Redundancy analysis (RDA) was conducted using CANOCO (Version 5, Microcomputer Power). Core species were defined as the species that were present in all sampled layers, and specific species were defined as the species that were only present within a specified water zone. The circos analysis for core species was done via online tool on http://circos.ca/.

## Results

### Environmental Conditions and Cellular Abundance

**FIGURE 1:**
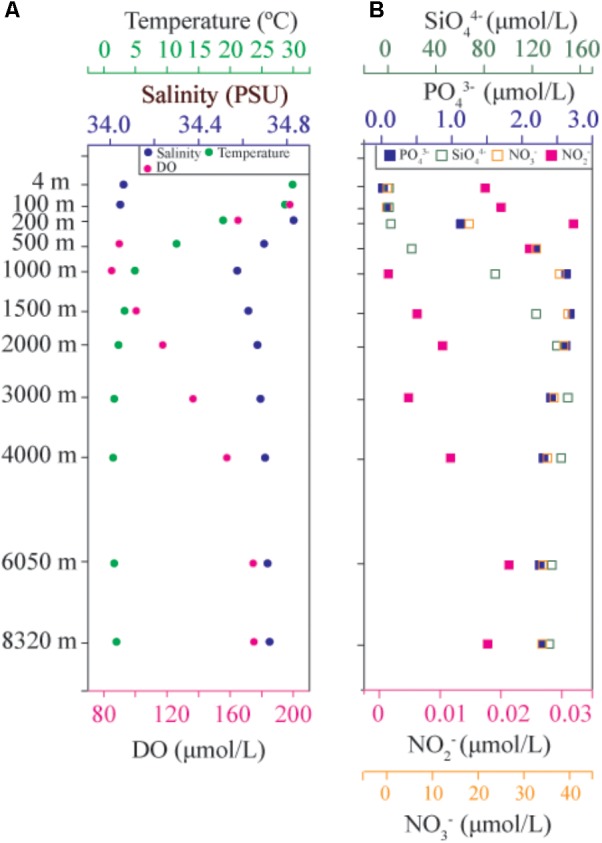
Vertical distribution patterns of environmental parameters. **(A)** Temperature, salinity, DO, and pH; **(B)** Nutrient concentrations, including PO_4_, NO_3_, NO_2_, and SiO_4_.

A total of 11 water samples were collected from depths 4–8320 m at the same location (11.38°N, 142.41°E, **Supplementary Table S1**), and these included samples from the epipelagic (Epi; 4 and 100 m), mesopelagic (Mes; 200 and 500 m), bathypelagic (Bat; 1000, 1500, 2000, 3000, and 4000 m), and hadal (6050 and 8320 m) zones. The temperature gradually decreased from the surface to the hadal zone, and these results were similar to those of a previous observation (Nunoura et al., 2015). The salinity ranged from 34.06 to 34.83 PSU, the highest salinity was found at 200 m (**Figure 1A**). The DO concentration was 197.50 μM/L at 100 m, and DO decreased to 84.92 μM/L at 1000 m, where an oxygen minimum zone exists and was firstly reported by Nunoura et al. (2015); then DO gradually increased with the increase in depth. The DO concentrations of the hadal zone, which were 174.40 and 174.80 μM/L at 6050 and 8320 m, respectively, were lower than those of the Epi zone (**Figure 1A**). The concentration of nitrate (NO_3_^-^) was 0.09 μM/L at 4 m, and it gradually increased to a maximum concentration of 39.35 μM/L at 1500 m before it decreased to 34.00 μM/L at 8320 m (**Figure 1B**). The concentration of nitrite (NO_2_^-^) was less than 0.4 μM/L throughout the water column. The distribution pattern of the PO_4_ concentration was similar to that of nitrate and nitrite. The phosphate (PO_4_^3-^) concentration was 0.018 μM/L at 4 m, and it reached a maximum concentration of 2.70 μM/L at 1500 m before it slightly decreased to 2.28 μM/L at 8320 m (**Figure 1B**). The concentration of silicate (SiO_4_^4-^) ranged from 0.55–149.61 μM/L, and the maximum concentration was detected at 3000 m (**Figure 1B**); furthermore, the silicate concentration of the Epi zone was higher than that of the other zones.

**FIGURE 2:**
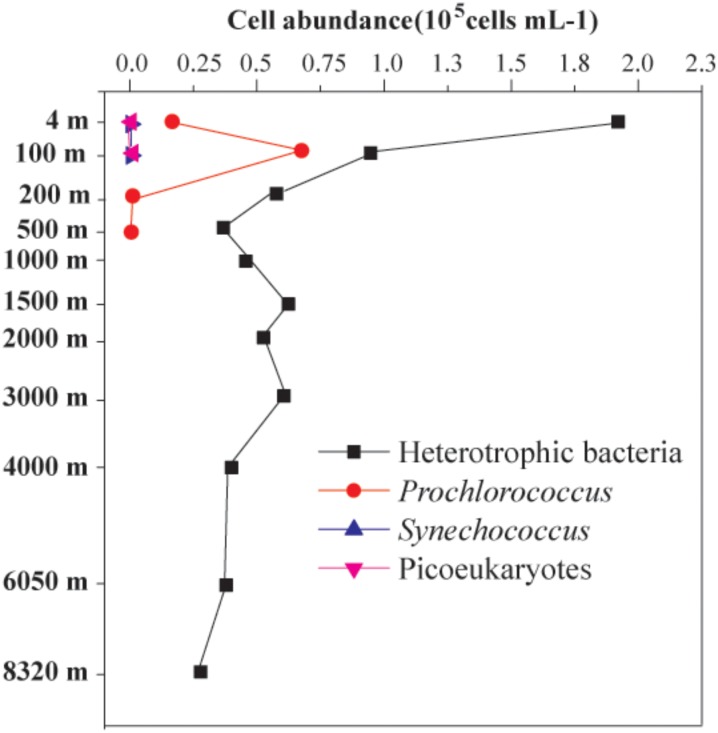
Vertical distribution patterns of the abundances of *Prochlorococcus, Synechococcus*, picoeukaryotes, and heterotrophic bacteria.

The cellular abundance of PN were measured based on the differential cell types, including heterotrophic bacteria, *Prochlorococcus, Synechococcus*, and picoeukaryotes, by using flow cytometry. The maximum (1.92 × 10^5^ cells mL^-1^) and minimum (2.78 × 10^4^ cells mL^-1^) abundances of heterotrophic bacteria were found at depths of 4 and 8320 m, respectively. The abundance of *Prochlorococcus* was 1.66 × 10^4^ cells mL^-1^ and 6.74 × 10^4^ cells mL^-1^ at the depths of 4 and 100 m, respectively (**Figure 2**). However, the *Prochlorococcus* from the other nine layers (i.e., 200–8320 m) and the *Synechococcus* and abundance of picoeukaryotes from all 11 layers were less than 1.0 × 10^4^ cells mL^-1^.

### Alpha-Diversity Patterns

**FIGURE 3:**
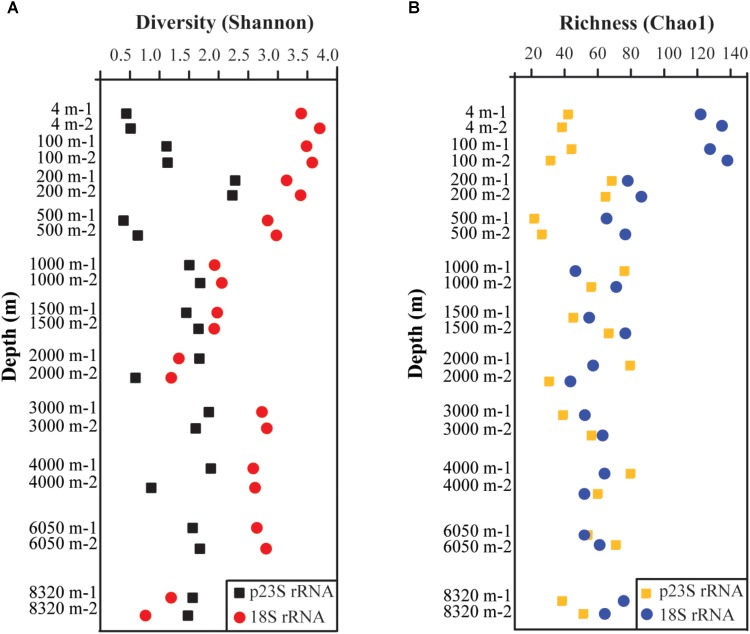
Vertical distribution patterns of diversity (Shannon) **(A)** and richness (Chao1) **(B)** of 18S and p23S rRNA gene analysis for PN.

The phytoplankton richness and diversity were evaluated using the Chao1 and Shannon indexes, respectively (**Figure 3**). The diversity pattern revealed by the 18S rRNA was quite different than that revealed by the p23S rRNA (**Figure 3A**). The 18S rRNA analysis showed that the duplicates of each layer were very similar, but the p23S rRNA analysis showed a slightly different pattern. In the p23S rRNA gene analysis results, both the 2000 and the 4000 m samples displayed large differences between their duplicates, which might have been caused by sequencing bias and/or sampling. As previously reported, the technical reproducibility of the amplicon sequencing-based detection technique is quite low; however, amplicon sequencing-based detection is still useful in analyzing microbial community structure (Zhou et al., 2011). Furthermore, because we performed two sequences, there should be an increase in the confidence of our results. The highest diversity of the 18S rRNA gene was found at 4 m, and the highest diversity of the p23S rRNA gene was found at 200 m. The richness of the Epi zone was relative higher than the richness values of the other zones, as revealed by the 18S rRNA analysis; however, the highest richness revealed by the p23S analysis was in the Mes zone (**Figure 3B**). Interestingly, the community diversity and richness values of the samples from 8320 m (i.e., the hadal zone) were not the lowest.

### Pico- and Nano-Phytoplankton in the Mariana Trench

**FIGURE 4:**
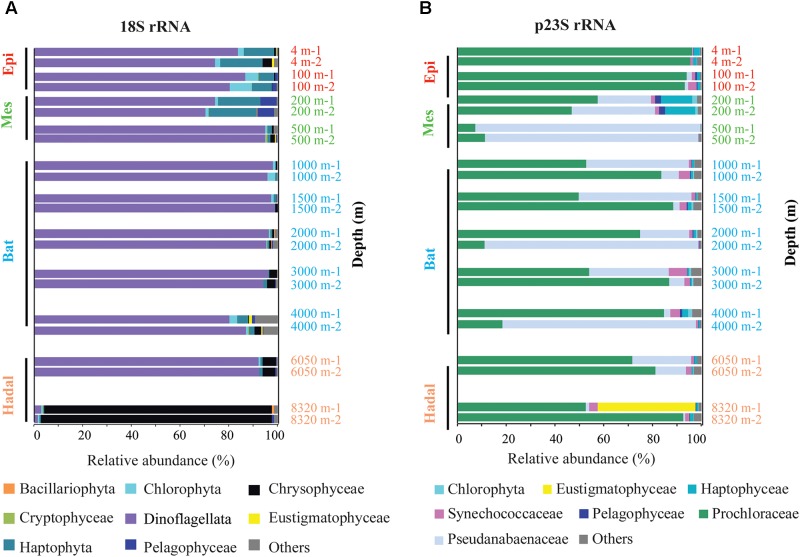
Vertical distribution patterns of PN community composition in the Challenger Deep. **(A)** 18S rRNA gene assemblage and **(B)** plastid 23S rRNA gene assemblage. “1” and “2” at the end of depth were used to distinguish the duplicates.

The results of the 18S rRNA analysis showed that the compositions of the duplicates from each layer were very similar, and a total of 169 OTUs were identified as PN. These 169 OTUs belonged to eight main phytoplankton groups (reads >0.1% in total), including Dinoflagellata (103 OTUs, 81.2% of total reads), Chlorophyta (13 OTUs, 1.5%), Haptophyta (22 OTUs, 4.6%), Cryptophyceae (3 OTUs, 0.1%), Chrysophyceae (11 OTUs, 10.0%), Bacillariophyta (3 OTUs, 0.1%), Eustigmatophyceae (3 OTUs, 0.1%), Pelagophyceae (1 OTU, 0.9%), and some other groups (reads <0.1% in total) (**Figure 4A**). The dinoflagellates were the dominant phytoplankton between the depths of 4 and 6050 m (**Figure 4A**), regardless of whether the relative abundance level or OTU level was used in the calculation; however, the dominant phytoplankton changed to Chrysophyceae at the depth of 8320 m.

The p23S rRNA analysis revealed 130 OTUs that belonged to phytoplankton, including species of cyanobacteria and other eukaryotic phytoplankton. Different from 18S rRNA, the p23S rRNA analysis results showed that the compositions of the duplicates from some layers were quite different, such as the samples from 1500, 2000, and 4000 m. The main differences were related to the cyanobacteria *Jaaginema* (OTU 92) and *Prochlorococcus* (OTU 8). The *Prochlorococcus*, which contributed to approximately 62.2% of the total reads, were the predominant genus in most of the layers. Haptophyta (2.0%), Synechococcaceae (2.1%), and Eustigmatophyceae (1.8%) were also identified with relative abundances higher than 1% of the total reads (**Figure 4B**). In addition, Pelagophyceae (0.4%) and Chlorophyta (0.4%) showed relative abundances higher than 0.1% but less than 1%, as determined by p23S rRNA analysis. At the OTU level, 46 OTUs and 15 OTUs contributed to Haptophyta and Chlorophyta, respectively, and Haptophyta was the most diverse group.

### Core and Specific Pico- and Nano-Phytoplankton

**FIGURE 5:**
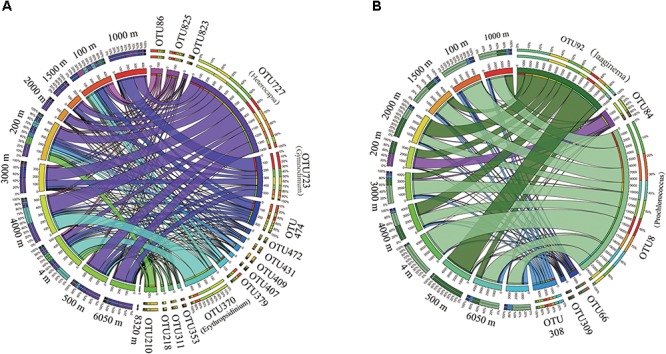
The distribution of core OTUs along the water column in the Mariana Trench. **(A)** 18S rRNA gene assemblage, the OTUs with relative abundances >0.1% were used in this analysis and **(B)** plastid 23S rRNA gene assemblage, the OTUs with relative abundances >0.1% were used in this analysis. The annotation of each OTUs were listed in the **Supplementary Table S2**.

To identify the core and specific phytoplankton that were present in each water zone, the comparison analysis was constructed at the OTU level (**Supplementary Figure S1A** and **Supplementary Table S2**). The 18S rRNA gene analysis revealed 33 core OTUs present in all four zones. These OTUs contained species from Dinoflagellata (23 OTUs), Pelagophyceae (1 OTU), Chlorophyta (2 OTUs), Haptophyta (4 OTUs), Cryptophyceae (1 OTU), Bacillariophytina (1 OTU), and one uncultured species. Among core species, the OTU727 assigned to the dinoflagellate *Heterocapsa* displayed the highest relative abundance and was followed by OTU723, which was assigned to the dinoflagellate *Gymnodinium* (**Figure 5A**). Furthermore, 31 OTUs, 6 OTUs, 15 OTUs, and 6 OTUs specifically belonged to the Epi, Mes, Bat, and hadal zones, respectively. For the OTUs that were specific to the hadal zone, the specific species were assigned to Chrysophyceae, Dinoflagellata, Bacillariophyta, and Haptophyta.

In the p23S rRNA gene analysis, 27 core OTUs were identified, and they were assigned to Haptophyta (7 OTUs), Pelagophyceae (1 OTU), *Prochlorococcus* (11 OTUs), *Synechococcus* (3 OTUs), Chlorophyta (1 OTU), and one unranked eukaryote (**Supplementary Figure S1B** and **Supplementary Table S2**). In all of these core OTUs, OTU8 (*Prochlorococcus*) [followed by OTU92 (*Jaaginema*)] was the most dominant OTU in all of the four zones (**Figure 5B**). For the specific OTUs, 8 OTUs, 11 OTUs, 21 OTUs, and 5 OTUs were only present in the Epi, Bat, Mes, and hadal zones, respectively. The OTUs that were specific to the hadal zone included *Coccomyxa* (Chlorophyta), *Chrysochromulina* (Haptophyta), *Ochromonas* (Chrysophyceae), and *Synura* (Synurophyceae).

### Relationships Between Pico- and Nano-Phytoplankton and Environmental Factors

**FIGURE 6:**
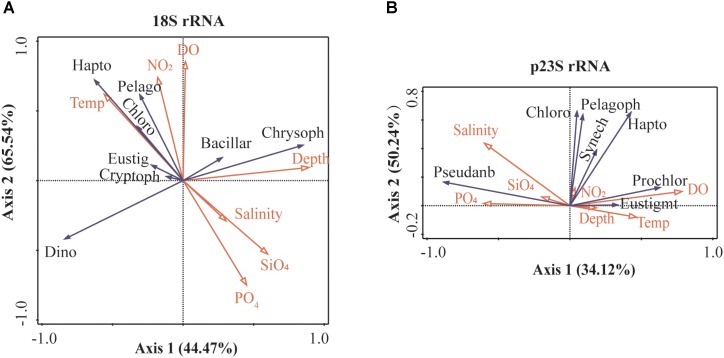
Redundancy analysis (RDA) diagram illustrating the relationship between PN community and environmental factors in the Mariana Trench. **(A)** 18S rRNA gene assemblage and **(B)** plastid 23S rRNA gene assemblage. The OTUs with relative abundances >0.01% were used in this analysis. Bacillar, Bacillariophyta; Chloro, Chlorophyta; Chrsop, Chrysophyceae; Crypto, Cryptophyceae; Dino, Dinoflagellata; Eustig, Eustigmatophyceae; Hapto, Haptophyta; Pelago, Pelagophyceae; Prochlor, Prochloraceae; Pseudanb, Pseudanabaenaceae; and Synechoc, Synechococcaceae.

RDA was employed to assess the relationships between the PN and environmental factors. The environmental factor correlation analysis showed that PO_4_ and NO_3_ had a positive relationship; thus, only the PO_4_ was selected for further analysis. According to the RDA across all the 18S rRNA PN communities, four environmental factors, including depth, PO_4_, salinity, and temperature, significantly contributed to the variation in the PN communities; in contrast, NO_2_, SiO_4_, and DO had almost no correlation with the distribution of PN communities (**Supplementary Table S3**). RDA (**Figure 6A**) showed that the first axis explained 44.47%, while the first two axes explained 65.54% of the total variation in the relative abundance of the 18S rRNA communities and 76.50% of the cumulative variation in the 18S rRNA communities and environmental factors. Chrysophyceae and Bacillariophyta were positively correlated with depth, while some groups, such as Chlorophyta and Haptophyta were significant negatively correlated with depth. Haptophyta, Chlorophyta, and Pelagophyceae were negatively correlated with salinity and PO_4_; however, they were positively correlated with temperature.

RDA explained 63.10% of the cumulative variation in the p23S rRNA communities and environmental factor relationships; furthermore, RDA showed that DO, depth, and salinity significantly contributed to the variation in the PN communities, while the other environmental factors had almost no correlation with the distribution of the PN communities (**Supplementary Table S3**). The results (**Figure 6B**) showed that the first RDA axis explained 34.12% of the total variation in the p23S rRNA communities. The first two RDA axes explained 50.24% of the total variation in the relative abundance of the p23S rRNA communities. Chlorophyta, Pelagophyceae, and Pseudanabaenaceae were negatively correlated with depth, and Chlorophyta and Pseudanabaenaceae were positively correlated with salinity. Haptophyta and Prochloraceae were positively correlated with DO.

In addition, the 18S and p23S rRNA communities were combined together, and their correlations with the environmental factors were analyzed (**Supplementary Table S3**). The results indicated that the depth, which was followed by temperature, was the most important environmental factor in terms of structuring the vertical distribution of PN community in the Mariana Trench. The temperatures were gradually decreased with increasing depth (**Supplementary Table S1**). RDA (**Supplementary Figure S2**) showed that the first axis explained 36.83%, while the first two axes explained 55.03% of the total variation in the relative abundance of the 18S rRNA + p23S rRNA communities and 68.2% of the cumulative variation in the 18S rRNA + p23S rRNA communities and environmental factors. Chrysophyceae, Eustigmatophyceae, Synechococcaceae, and Prochloraceae were positively correlated with depth, while Dinoflagellata, Chlorophyta, Haptophyta, and Pseudanabaenaceae were significant negatively correlated with depth. Haptophyta, Dinoflagellata, and Prochloraceae were significant positively correlated with temperature, while Chrysophyceae, Pseudanabaenaceae, Eustigmatophyceae, Cryptophyceae, and Bacillariophyta were negatively correlated with temperature. In addition, the nutrients (PO_4_, NO_3_, and NO_2_) and salinity also significantly contributed to the structure of the PN communities.

## Discussion

As important players in the oceanic biological carbon pump processes, phytoplankton contribute to the export of carbon from the surface of the ocean to the deep ocean (Richardson and Jackson, 2007; Tréguer et al., 2017; Leblanc et al., 2018), and may even provide an important source of energy to organisms present in the hadal trench. In this study, the diversity and composition of the PN present in the Challenger Deep were examined by 18S and p23 rRNA gene analysis. Compared to single gene analysis, the two target gene analysis methods potentially offer a more comprehensive view of the phytoplankton diversity. Both cyanobacteria and various eukaryotic PN communities were identified from the hadal trench. The vertical distribution pattern identified here suggested that the PN communities have important roles in the process of exporting organic matters from the surface to the deep sea in the Mariana Trench, and these results improved the understanding of the biological carbon pump of the hadal trench.

### Environmental Factors Controlling the Mariana Trench Pico- and Nano-Phytoplankton

The RDA revealed that the depth, temperature, salinity, and nutrients (PO_4_, NO_3_, and NO_2_) might be the significant environment factors that structure the PN community in the Mariana Trench. Abiotic environmental factors, such as salinity, depth, temperature, light, and nutrients, can significantly influence the physiological and ecological characteristics of phytoplankton (Chen et al., 2014; Liang et al., 2016; Gittings et al., 2018; Wei et al., 2018). In addition, some unexpected results were found, such as the positive relationships between depth and Chrysophyceae and between depth and Eustigmatophyceae. Similarly, the Chrysophyceae were also predominant microbial eukaryotes at the depth of 8727 m of the Challenger Deep (Xu et al., 2018). These results suggested that Chrysophyceae and Eustigmatophyceae may sink to the deep sea more easily than other phytoplankton groups, and/or they possess special biological characteristics that help them resist the extreme conditions, such as the cold temperature and high hydrostatic pressure. For example, a high relative abundance of the Eustigmatophyceae *Nannochloropsis* was found at 8320 m. *Nannochloropsis* species are capable of accumulating a large amount of lipids, which may help them adapt to low temperatures (Murakami et al., 2018). For Chrysophyceae, the most OTUs were assigned to the genus *Paraphysomonas*, which include the heterotrophic and potentially low-temperature resistant species (Rose et al., 2009).

Moreover, other environmental factors and accidental events, such as currents, turbulence, and earthquakes, were not included in the analysis; however, these factors could also affect the phytoplankton community in the Mariana Trench.

### The Composition of the Cyanobacteria Community in the Mariana Trench

In this study, the sampling site that belonged to the oligotrophic zone and was located at a low latitude had a small population of *Synechococcus* (i.e., less than 1000 cells at 4 and 100 m), and its abundance was considerably less than that of *Prochlorococcus*. The results were similar to previous findings that stated *Prochlorococcus* was most abundant at the surface (i.e., 0–100 m) and decreased with depth (Johnson et al., 2006); furthermore, its abundance corresponded to previous reports in which it ranged from 2.8 × 10^3^ to 4.4 × 10^4^ cells mL^-1^ (Flombaum et al., 2013). Both *Prochlorococcus* and *Synechococcus* are prokaryotic picophytoplankton, but their distribution patterns were quite different. *Synechococcus* are vitally ubiquitous in all oceans, and they are more abundant in the eutrophic zone than in the oligotrophic zone; in contrast, *Prochlorococcus* are less ubiquitous and much more commonly distributed in low latitudes (40°N to 40°S), and they extend much deeper in the water column, with a common maximum concentration at depths of ∼150–200 m (Partensky et al., 1999; Johnson et al., 2006). The temperature and light (but not the nutrients) were the factors that had the most control over the distribution of *Prochlorococcus* and *Synechococcus* (Flombaum et al., 2013).

Interestingly, *Jaaginema*, one type of filamentous cyanobacterium, was found by the p23S rRNA analysis, and they were abundant from 200 to 6050 m. In general, the genus *Jaaginema* is mainly present in freshwater ecosystems; however, several species have been identified in marine ecosystems, including *Jaaginema litorale* (Brito et al., 2017). The species identified from this study corresponded to the previously described marine species *Jaaginema litorale*. Most studies of this genus have been based on morphology, and only very few sequences have been recorded in the database, which may explain why they have rarely been identified in oceans in previous studies that used gene analysis-based methods.

### The Composition of Eukaryotic Pico- and Nano-Phytoplankton in the Mariana Trench

The abundance of eukaryotic PN was relatively low, i.e., less than 2000 cells mL^-1^. Nevertheless, the 18S rRNA and p23S rRNA gene analyses revealed the diversity of eukaryotic PN due to the high sensitivity of this molecular method. The phytoplankton structure was dependent on the seasons, geographical location, and environmental nutrient conditions (de Vargas et al., 2015; Kataoka et al., 2017), and the physical conditions, such as turbulence, mixed layer depth and thermal stratification, were also important regulators of phytoplankton abundance (Barton et al., 2015). Generally, diatoms and dinoflagellates are the most dominant phytoplankton in the world’s ocean. However, the dinoflagellates were among the predominant PN, whereas diatoms were rare in this study. Diatoms are often dominant phytoplankton in eutrophic regions; moreover, diatoms are very rarely found in mesotrophic regions, and no significant relative abundance has been found in oligotrophic regions (Shi et al., 2011). These data corresponded to the traditional views of dinoflagellate and diatom ecology. In the traditional view, these two phytoplankton are generally distributed in differential niches; the diatoms prefer turbulent, nutrient-rich conditions, while the dinoflagellates prefer calm, nutrient-poor conditions (Irwin et al., 2012; Barton et al., 2015). Moreover, since we have only analyzed the PN communities, the results were also similar to previous findings that the population of diatoms (pico- and nano-size) were smaller than that of dinoflagellates in the sunlit waters of the global oceans (de Vargas et al., 2015).

In addition, the dinoflagellates, as eukaryotes, were the dominant PN according to the 18S rRNA gene analysis. The high copy numbers of some dinoflagellates species 18S rRNA genes may contribute to their relative high abundance in 18S rRNA gene communities (Guo et al., 2016). Few dinoflagellate OTUs were found using the p23S rRNA gene analysis, and the complex genetic feature of dinoflagellate plastids may explain these results, i.e., the multiple origins of plastid during evolution resulted in the genetic divergence of dinoflagellate plastids (Zhang et al., 2000; Xia et al., 2013; Yamada et al., 2017). Furthermore, the dinoflagellates are very diverse and include autotrophs (photosynthetic), heterotrophs, and mixotrophs. All photosynthetic and mixotrophic dinoflagellates contain the plastid, while the heterotrophic dinoflagellates lost the most of the plastid genes (Saldarriaga et al., 2001). In the taxonomic analysis, since it was difficult to classify the OTUs at the species level, some of the heterotrophic dinoflagellates could be included in the final analysis.

The Haptophyta, of which most of them were Prymnesiophyceae, were identified as one of the dominant picophytoplankton in the Epi and Mes zones. The molecular methods confirmed that Prymnesiophyceae were present in the Pacific Ocean, Atlantic Ocean, and Indian Ocean (Shi et al., 2011; Kirkham et al., 2013), suggesting they have a wide geographic distribution, especially in most mesotrophic and oligotrophic regions (Fuller et al., 2006; Lepere et al., 2009; Jardillier et al., 2010; Shi et al., 2011). Moreover, the relatively high abundance of pico-Prymnesiophyceae were found in the Pacific Ocean, Atlantic Ocean, and Indian Ocean (Kirkham et al., 2013), and the *Tera* ocean also had a relatively high abundance of Haptophyta (de Vargas et al., 2015).

In addition, the OTUs assigned to Chlorophyta, Pelagophyceae, Synurophyceae, Eustigmatophyceae, and Chrysophyceae were also identified, but their relative abundances were very rare. These phytoplankton are widespread in the world’s oceans, but their distribution characteristics are different. Pelagophyceae were generally present in the open ocean or/and the oligotrophic region as well as the mesotrophic region, while the Chlorophyta were mostly distributed in the coastal ocean or/and the eutrophic region (Not et al., 2008; Shi et al., 2011; Balzano et al., 2012; Kataoka et al., 2017). Moreover, Pelagophyceae and Chlorophyta are not generally the dominant phytoplankton in most oceans (Kirkham et al., 2011, 2013; Shi et al., 2011; de Vargas et al., 2015; Kataoka et al., 2017). Chrysophyceae showed similar geographic distribution patterns as Prymnesiophyceae at a global scale; however, Chrysophyceae were detectable at deeper depths of the water column (Kirkham et al., 2013). Synurophyceae and Eustigmatophyceae have been detected sporadically in the open ocean in previous studies (Kirkham et al., 2011, 2013; Pernice et al., 2016; Xu et al., 2017).

### The Presence of Pico- and Nano-Phytoplankton in the Deep Sea and Hadal Trench

Interestingly, many PN were found in the deep sea, including the hadal zone; moreover, some OTUs were found in the Bat and hadal zones but were not found in the upper layer zones. This was not the first time phytoplankton have been detected in the deep-sea/dark ocean. Living phytoplankton cells, including dinoflagellates, diatoms, and cyanobacteria, were observed at depths of 2000–4000 m using a microscope and/or flow cytometry analysis (Jiao et al., 2013; Agusti et al., 2015). The phytoplankton Dinoflagellata, Bacillariophyceae, Chrysophyceae, Prymnesiophyceae, Prasinophyceae, and Dictyochophyceae were detected in the water column at depths of 3000–4000 m using 18S rRNA analysis (Pernice et al., 2016). Furthermore, some phytoplankton, such as Chrysophyceae, Synurophyceae, and Pelagophyceae, were also detected in the deep sea of the South China Sea (Xu et al., 2017). To date, the sinking mechanism is the most common and accepted theory accounting for the presence of phytoplankton cells in the deep sea (Jiao et al., 2013; Agusti et al., 2015). The sinking rate is one important factor of phytoplankton sinking mechanism (Huisman et al., 2002; Agusti et al., 2015; Leblanc et al., 2018). The sinking rate of micro-phytoplankton cells might reach to 124–732 m per day (Agusti et al., 2015), however, the sinking rate would be decreased with decreasing cell size (Richardson and Jackson, 2007). It indicated that the PN communities identified from hadal zone in this study might have presented in the dark for several weeks or several months. Furthermore, some of the PN cells from the hadal zone have been successfully cultivated in f/2 medium (**Supplementary Figure S3**), and the intact cells of cyanobacteria, dinoflagellates, and diatoms have been found in the deep sea at depths of 2000–4000 m (Jiao et al., 2013; Agusti et al., 2015). Moreover, the metabolic activity of phytoplankton did not decrease in the deep sea compared to that in the shallower waters (Xu et al., 2017). These data implied that the PN communities have survived for long time in the dark sea. There are at least three aspects that could explain this phenomenon. First, the sink rate should be much faster than expected if the sinking mechanisms account for this (Xu et al., 2017), the physical processes as well as biological processes, which could accelerate the sink rate, are involved in the transportation of phytoplankton to the deep sea (Jiao et al., 2013; Agusti et al., 2015). Second, the potential heterotrophic characteristic of phytoplankton may contribute to their long time survival in the dark sea. It is well known that many phytoplankton are mixotrophic (Stoecker et al., 2017), meaning they can obtain energy and/or nutrients through different methods under various environmental conditions. The heterotrophic phytoplankton could eat bacteria and/or large particulate matter via phagocytosis (Maruyama and Kim, 2013; Raven, 2013). In the present results, the predominant abundant phytoplankton communities at 8320, e.g., Eustigmatophyceae *Nannochloropsis* and Chrysophyceae *Paraphysomonas*, were reported to have heterotrophic characteristics. It indicated that heterotrophy is most likely a way of metabolism in the dark ocean. Third, some of phytoplankton cells in the deep sea might be in the resting stages, which could help phytoplankton cells present in the darkness for several decades or even up to 100 years (Ribeiro et al., 2011), and could survive again once be back to the suitable environments.

However, the sinking mechanism cannot explain our results, as some OTUs were found only in the Bat zone and the hadal zone, and these OTUs did not appear to have sunk from the vertical water layers. Physical processes such as currents and Ekman transport may contribute to these phytoplankton cells. Furthermore, the phytoplankton composition pattern in the hadal zone, especially at very deep depths (8320 m), was quite different from that in the shallower layers in this study (**Figure 4**). Distinct patterns and features were also revealed with other factors. For example, phototrophic pigments, including chlorophyll *a* and phaeophytin, were analyzed in the Challenger Deep, and the results revealed that the concentrations of these pigment were highest in the Challenger Deep compared to the shallower waters, implying that the pigment in the Challenger Deep was younger, more labile and possibly more nutritious than those at the shallower reference site (58 km from the trench and approximately 6000 m in depth) (Glud et al., 2013). The bacterial community was different from those of the shallower zones, and the chemolithotrophic populations of the hadal zone were more abundant in the upper abyssal waters (Nunoura et al., 2015); furthermore, this unique community formation could not be explained by the vertical flux of sinking organic particles, but it may due to the endogenous recycling of organic matter in the hadal waters (Nunoura et al., 2015). In addition, biomass generally decreases with increases in depth in the deep sea (Rex et al., 2006); however, this pattern may be different in the hadal zone (Glud et al., 2013). All of these data suggested that the general distribution pattern of organisms in the oceans could not be applied to the hadal zone, and the distinct geomorphology of the hadal zone may contribute to the unique organism composition characteristics.

**FIGURE 7:**
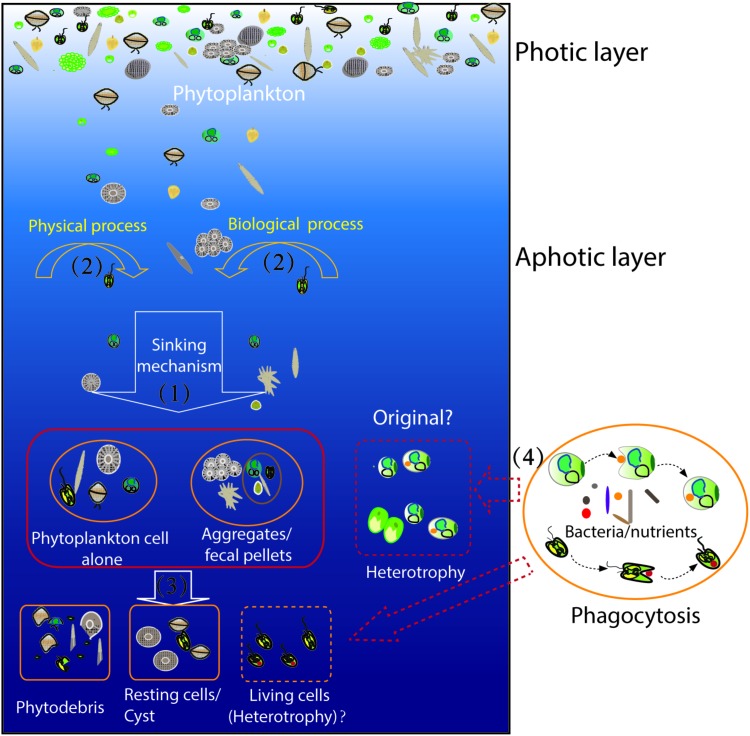
Schematic illustration of the mechanism of phytoplankton presence in the aphotic layer. The shown process/phenomenon marked within the solid-lined box indicate they have been identified; the marks within the dotted box indicate they were hypothesized. (1) Phytoplankton cells sank from aphotic layers into the aphotic layers of the sea; they could sink alone or within the aggregates/fecal pellets; (2)the physical process and/or biological process might be involved in the sinking process to accelerate cell sink; (3) the phytoplankton cells might be ultimately degraded into phytodetritus, or the cells might become resting cells or cysts; the cells also might live in the dark sea as heterotroph; and (4) the phytoplankton cells as heterotroph might prey on bacteria and/or large particulate matter to survive.

To date, studies on the phytoplankton of the deep sea are limited. It is very difficult to explain the presence and origins of the phytoplankton found in the deep sea. However, the relative abundances of phytoplankton in the deep sea could contribute to the transfer of carbon to the dark oceans, which would indicate they play important roles in the deep-sea carbon cycle. We hypothesized that the phytoplankton present in the deep sea (aphotic zone) could be separated into at least two types (**Figure 7**). One part of the phytoplankton cells came from the upper layer zones via fast sinking mechanisms. These cells may sink alone or within aggregates and/or zooplankton fecal pellets, and various physical processes could help these cells sink faster into the deep sea (Kriest and Evans, 2000; Turner, 2002; Jansen and Bathmann, 2007). These cells could live for several days, and they could ultimately be degraded into phytodetritus, and they also might become as resting cells or cyst. Another part of the phytoplankton cells could be heterotrophs that have been present in the deep sea for a long time. They may prey on bacteria and/or large particulate matter to survive.

## Conclusion

The present study for the first time reported the vertical distribution pattern of PN in the Mariana Trench. The dinoflagellates and the cyanobacteria *Prochlorococcus* were present throughout the entire water column. Some PN communities, which possess the heterotrophic characteristics, e.g., Eustigmatophyceae and Chrysophyceae, displayed higher relative abundance in the hadal zone. The presence of phytoplankton cells in the hadal zone may benefit from sinking mechanism and/or heterotrophic characteristics as well as resting characteristics of the cell. The present results highlighted the distinctive biosphere characteristics of the hadal trench environment and indicated that the phytoplankton could be involved in transportation of organic matters from euphotic to hadal zone, and some phytoplankton might survive with undetermined energy metabolism in the hadal zone.

## Author Contributions

RG performed the experiments. RG, YL, YX, and LW analyzed the data. CC and RX performed sampling. SM identified the phytoplankton cells. CZ and JT provided expedition oversight. CZ and JT participated in data analysis and discussion. YL and YZ designed the experiments. RG and YZ wrote the manuscript.

## Conflict of Interest Statement

The authors declare that the research was conducted in the absence of any commercial or financial relationships that could be construed as a potential conflict of interest.

## Footnotes

1 http://www.i-sanger.com

## Supplementary Material

FIGURE S1 Venn diagram of PN community composition from different ocean zones. (A) 18S rRNA gene assemblage; (B) plastid 23S rRNA gene assemblage. Epi, epipelagic; Bat, bathypelagic; and Mes, mesopelagic.

FIGURE S2 Redundancy analysis (RDA) diagram illustrating the relationship between PN community (18S rRNA + plastid 23S rRNA gene assemblages) and environmental factors in the Mariana Trench. The OTUs with relative abundances >0.01% were used in this analysis. Bacillar, Bacillariophyta; Chloro, Chlorophyta; Chrsop, Chrysophyceae; Crypto, Cryptophyceae; Dino, Dinoflagellata; Eustig, Eustigmatophyceae; Hapto, Haptophyta; Pelago, Pelagophyceae; Prochlor, Prochloraceae; Pseudanb, Pseudanabaenaceae; and Synechoc, Synechococcaceae.

FIGURE S3 Image of *Chaetoceros* sp. isolated from the hadal zone (depth, 8320 m) of Mariana Trench.

TABLE S1 Co-ordinates and environmental data.

TABLE S2 Annotation of core, specific bathypelagic, and specific Hadal OTUs by 18S rRNA and plastid 23S rRNA gene analysis.

TABLE S3 Explains and contribution value of each evironmental factor (18S rRNA + p23S rRNA).
