# Supplementary figures and images for: Insight Into the Pico- and Nano-Phytoplankton Communities in the Deepest Biosphere, the Mariana Trench
Source: Front Microbiol. 2018 Sep 26;9:2289. doi: 10.3389/fmicb.2018.02289 (PMC6168665; doi:10.3389/fmicb.2018.02289)

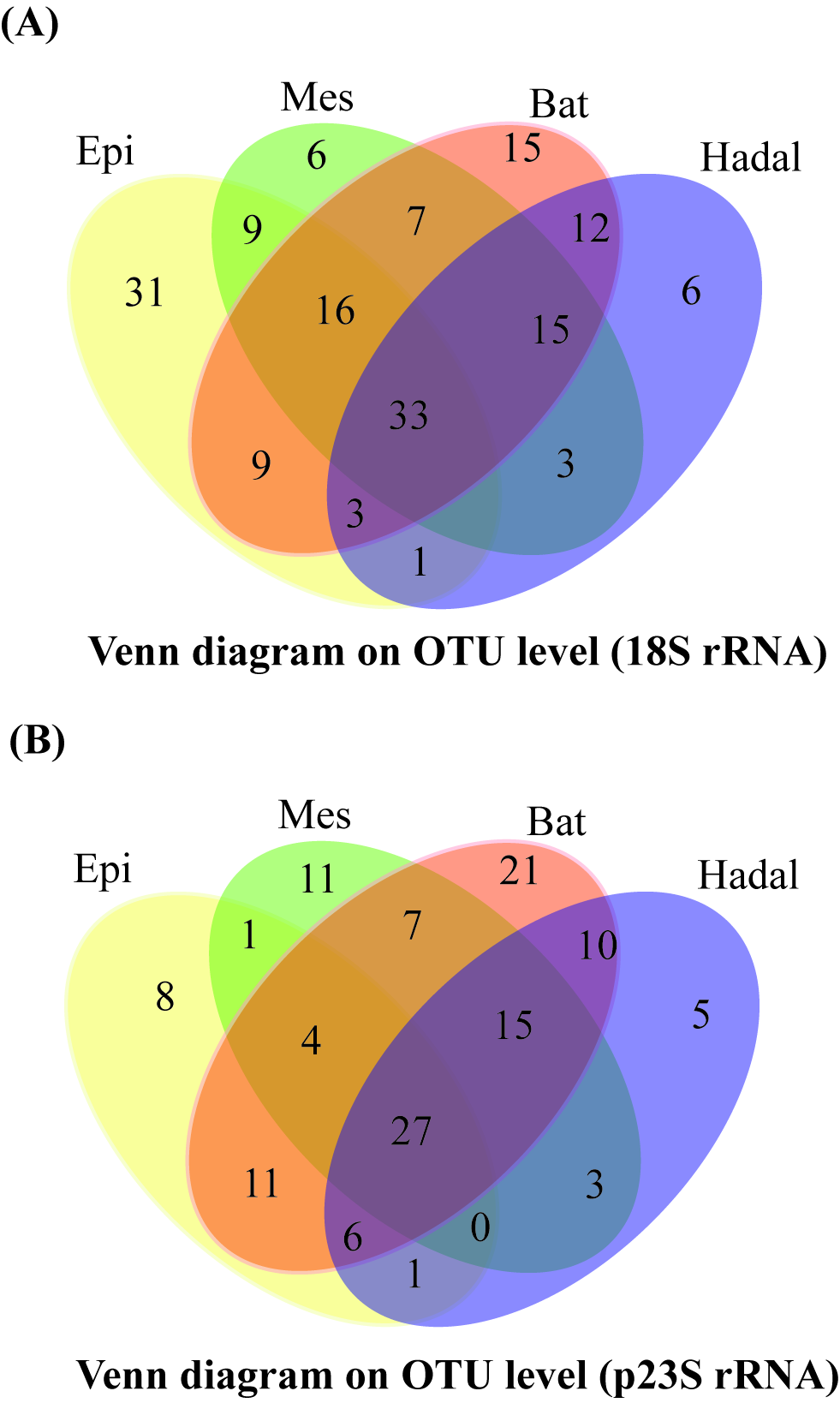

Supplement: FIGURE S1 — Venn diagram of PN community composition from different ocean zones. (A) 18S rRNA gene assemblage; (B) plastid 23S rRNA gene assemblage. Epi, epipelagic; Bat, bathypelagic; and Mes, mesopelagic. [file Image_1.TIF]

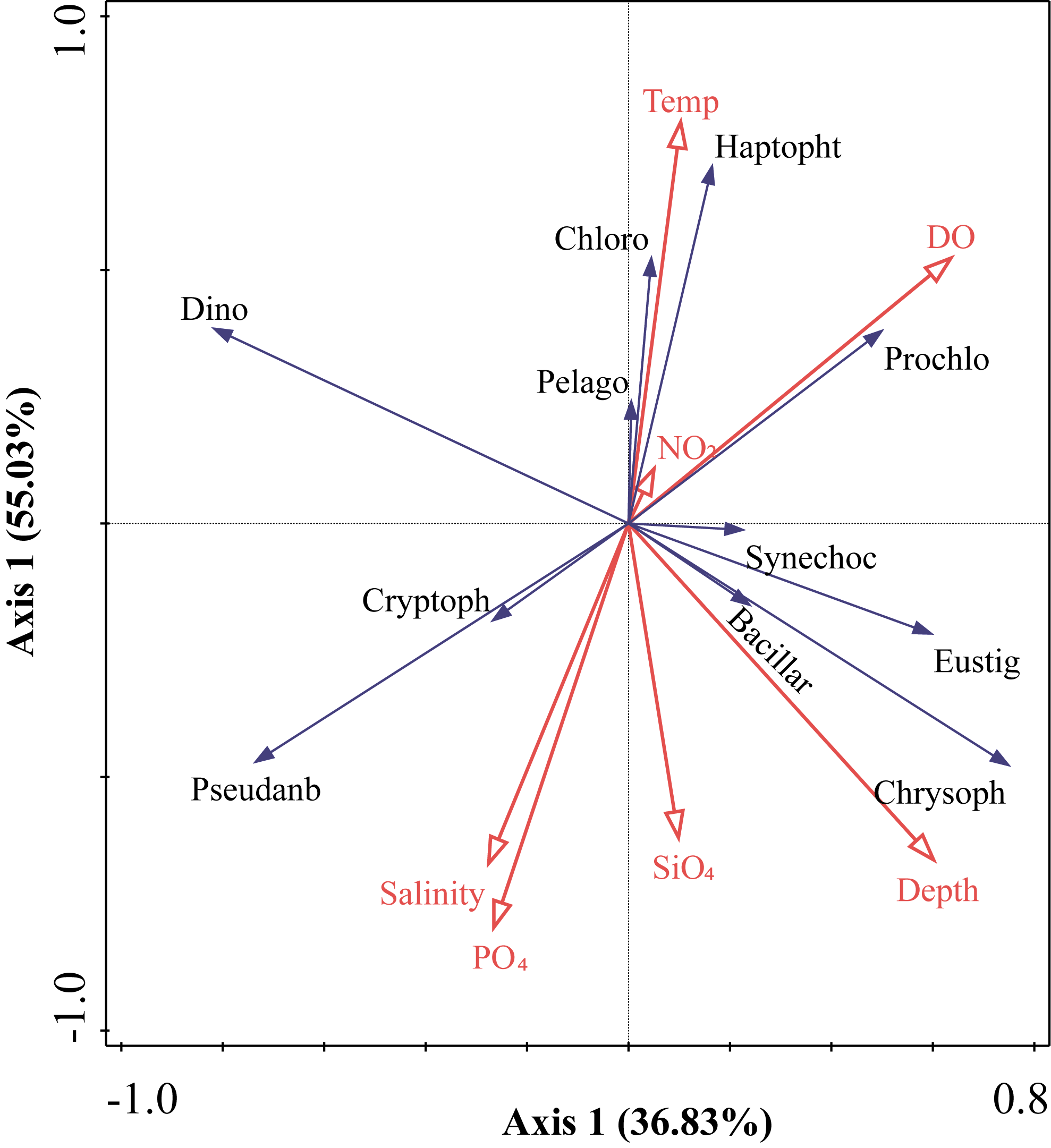

Supplement: FIGURE S2 — Redundancy analysis (RDA) diagram illustrating the relationship between PN community (18S rRNA + plastid 23S rRNA gene assemblages) and environmental factors in the Mariana Trench. The OTUs with relative abundances >0.01% were used in this analysis. Bacillar, Bacillariophyta; Chloro, Chlorophyta; Chrsop, Chrysophyceae; Crypto, Cryptophyceae; Dino, Dinoflagellata; Eustig, Eustigmatophyceae; Hapto, Haptophyta; Pelago, Pelagophyceae; Prochlor, Prochloraceae; Pseudanb, Pseudanabaenaceae; and Synechoc, Synechococcaceae. [file Image_2.TIF]

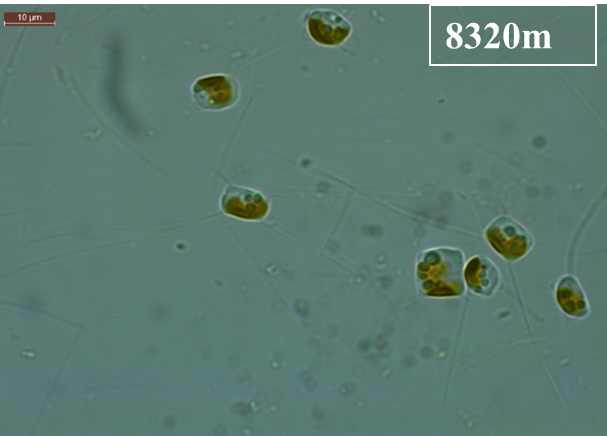

Supplement: FIGURE S3 — Image of Chaetoceros sp. isolated from the hadal zone (depth, 8320 m) of Mariana Trench. [file Image_3.PNG]
